# Employee workspace preferences in a mandated hybrid work policy: A discrete choice experiment

**DOI:** 10.5271/sjweh.4264

**Published:** 2026-03-01

**Authors:** Emmanuel Aboagye, Willings Botha, Helena Tinnerholm Ljungberg, Christina Bodin Danielsson, Irene Jensen

**Affiliations:** 1 Unit of Intervention and Implementation Research for Worker Health, Institute of Environmental Medicine, Karolinska Institutet, Stockholm, Sweden.; 2 Department of Psychology, Norwegian University of Science and Technology, Trondheim, Norway.; 3 Novo Nordisk, Medical & Science Patient Focused Drug Development, Oxford, United Kingdom.; 4 Construction Management, Architecture and Civil Engineering, Chalmers University of Technology, Chalmers University of Technology, Gothenburg, Sweden.; 5 School of Architecture, Royal Institute of Technology (KTH), Stockholm, Sweden.

**Keywords:** choice experiment, desk sharing, higher education, office design, operational support staff, personalization

## Abstract

**Objectives:**

Understanding employee workspace preferences is crucial for designing office work environments that meet their needs. This study investigated employee office design preferences within a mandated hybrid work model at a higher education institution.

**Methods:**

In this discrete-choice experiment (DCE), operational support staff (N=433) at a university participated in evaluating 12 pairs of hypothetical office design options, each varying across seven workspace attributes from a DCE survey. Preference weights indicating the relative strength of preference for each workspace design attribute level were used to calculate the importance of each attribute, conditional on the range of levels considered and relative to all other attributes included in the survey. The conditional relative importance of each attribute was calculated as the difference in preference weights for the most- and least-preferred level of that attribute. Subgroup analysis was performed on predefined, mutually exclusive subgroups, with results reported only for those exhibiting statistically significant differences in preferences.

**Results:**

The results showed that having a dedicated desk (ie, no desk sharing) was an important factor influencing preferences, followed by personalization and territoriality, opportunities for teamwork, and social interaction. Employees preferred having access to shared spaces for collaboration while also valuing dedicated desks for personal belongings. Private offices and quiet spaces were not strongly preferred. Preferences varied by demographic and work-related characteristics, including gender, age, commute distance, and home environment.

**Conclusions:**

This study shows that operational support staff in higher education prefer office designs that provide a dedicated desk, emphasize personalization, and social interaction. The strong preference for control over workspace and social connection highlights the office's role in supporting psychosocial well-being in flexible work arrangements. These findings are crucial for informing occupational health and safety strategies and designing workspaces that balance individual and collective requirements.

Flexible work arrangements (FWA) offer employees autonomy over where and when work is performed and have gained popularity since the pandemic (1). Studies indicate that employees who experienced teleworking during the pandemic are more inclined to continue teleworking afterward (2). However, many organizations are reluctant to implement solely teleworking, which is a limited option of FWA in certain occupations [eg, Gilson et al (3)], on a large scale. Consequently, these organizations aim to adopt flexible work practices, allowing individuals to work in a combination of external locations (eg, home, café, and work hubs) and the employer's premises (4).

The scale of this shift is substantial, making the optimization of flexible office design a pressing issue for a significant portion of the global workforce. In the United States and the European Union, for instance, it is estimated that many employees, potentially 20–40% of the workforce, could telework for a significant part of their week (5). This trend is particularly pronounced among occupational sectors characterized by administrative, support, and knowledge work, which are prevalent in public institutions, the service industry, and the technology sector. Therefore, understanding the office design preferences of these employees, a group that numbers in the tens of millions across Europe and North America alone, is crucial for creating work environments that support productivity, well-being, and retention on a massive scale.

In the FWA context, the impact of employee workspace perception and preference on workplace satisfaction must not be overlooked. Previous research shows that environmental satisfaction with ambient factors, noise, privacy, and design-related elements differ significantly between various office types (6). Employees' perceptions of how their workspace contributes to job satisfaction, pleasantness, and their ability to do a good job also vary across office designs (7). More broadly, office research identifies environmental disturbances (visual and acoustic) and stimuli (environmental and psychosocial) as central determinants of employee preferences (6). With the growing popularity of activity-based flexible offices (AFO), attention has increasingly turned to employee preferences within this specific design (8, 9). Findings highlight the importance of factors such as noise, workspace enclosure, and control in shaping workspace selection, with preferences differing by activity, yet full enclosure is not always desired even for work that requires concentration (10). Research on AFO environmental qualities has emphasized employee satisfaction, communication, collaboration, and productivity, showing that the availability of preferred workstations and alternative settings for uninterrupted work is especially valued (11). However, these spatial arrangements often require employees to move to supplementary spaces, and reluctance to switch workstations has been documented, [eg (12, 13),]. Despite the advances of AFO research, when it comes to understanding how physical work environment attributes shape preferences for other office designs in hybrid and flexible work arrangements, the evidence base is still scarce.

Furthermore, previous research has shown that workplace preferences are highly context-dependent, with employees valuing different attributes depending on whether they work from home or in the office (14). AFO have further demonstrated that office design is not a one-size-fits-all solution, as knowledge workers consistently emphasize the need to balance collaboration opportunities with spaces that support concentration and psychological comfort (10). Flexible work choices are also shaped by a weighing of home versus office affordances, underscoring the need for organizations to optimize both environments rather than favoring one (15). Moreover, perceived office quality acts as a push–pull factor influencing teleworking preferences, with poor office conditions driving employees away and high-quality conditions drawing them back (16). A survey-based experiment identified two employee segments in flexible work: one that wants to return to the office and one that prefers to work from home (17). Crowdedness and the availability of private spaces for concentration and meetings influenced the decision to work from the office. Despite these advances, less is known about how employees prioritize specific workspace attributes – such as territoriality, personalization, and spaces that give opportunities for social connection – in hybrid and post-pandemic contexts, and how such preferences might translate into practical design and management strategies.

The impact of the pandemic on working arrangements continues to evolve as does its impact on the accelerated adoption of FWA. Existing literature indicates that FWA, including hybrid work arrangements, are an ambivalent experience (18). The increasing prevalence of remote work has prompted many organizations to re-evaluate their operational models, debating the merits of fully remote versus hybrid approaches. The hybrid work model has emerged as a modern approach, accommodating employees working both from the employer's premises and alternative external locations. Hybrid work will likely be a prominent feature of the modern office landscape. Further research is needed to investigate the impact of the increasing prevalence of hybrid office models in modern work environments.

This study investigated how a hybrid work policy influences employee office design preferences, including physical and social characteristics of an on-site office environment at a higher education institution. It informs flexible work policies by identifying employee preferences for office workspace attributes, enabling organizations to better meet employee needs. Furthermore, it expands the understanding of how spaces that promote social relations in the office environment influence workspace preferences in a hybrid work context and examines the attributes that are associated with office attendance. The findings offer practical guidance for organizations adapting workspaces for hybrid working arrangements.

## Methods

This study was part of an organization-wide prospective cohort project called “Future Work”. The primary aim of the Future Work project was to follow up on the development of the work environment, including leadership, health, and performance, during the implementation of a hybrid working arrangement at a higher education institution. The cohort was followed with repeated measures from August 2021 to April 2023.

### Study design

A cross-sectional web-based discrete-choice experiment (DCE) survey was developed and distributed to all staff members in the cohort (administrative support services at a higher education institution) in January 2023 over three months. During this period, the organization mandated a hybrid work policy, such that employees could work up to 49% from home. The Swedish Ethical Review Authority approved the Future Work project (registration number 2021-03637). All procedures complied with relevant laws and institutional guidelines approved by the appropriate committees.

### Discrete-choice experiment

The DCE is a commonly used method to investigate individuals' preferences and decision-making processes (20). The approach suggests that individuals engage in decision-making by carefully considering and weighing trade-offs among available alternatives to select the most advantageous option. The DCE study design and analyses were developed following good research practice guidelines (21, 22).

The attributes used in the DCE survey were selected to quantify the relative importance of office workspace characteristics in influencing employee preferences. Using four criteria, atttributes were selected: (i) based on their proven significance in prior office design research, ensuring alignment with established findings on workspace satisfaction, health, and performance (23, 24); (ii) if they aligned with the specific research aims of this project, namely, to examine how office design features affect preferences for operational support staff; (iii) for their clarity and feasibility in a stated-choice context, ensuring that they could be coherently described and evaluated by respondents (25); (iv) if they were assessed to ensure that inclusion would not impose an excessive burden on respondents, maintaining the practicality of the experiment (26).

An initial set of workspace attributes and levels was chosen after reviewing the literature and consulting with experts. The experts included researchers, occupational health professionals, an architect, and administrative employees, who ranked characteristics – such as office design, office setting, working conditions, service function and support at the office – and workplace activity and outcomes, which are crucial for a functional office environment. This collaborative approach ensured that the selected attributes were grounded in both empirical evidence and expert knowledge.

The final seven attributes and their levels are presented in table 1, these reflect central dimensions of the office environment emphasized in previous research: (i): *office type/design*, linked to differences in satisfaction, health, and well-being between cell offices, open-plan offices, and activity-based offices (23, 24); (ii) *desk sharing*, which is a key aspect of non-territorial working and has been shown to influence satisfaction, productivity, and health. These effects are often linked to a complex interplay of factors, including the loss of desk ownership, reduced personalization, and disruptions to daily routine (24, 27); (iii) *privacy and quiet workspace*, a well-documented factor in supporting concentration, satisfaction, and job performance (28); (iv) *personalization and territoriality*, reflects how personalization is described in workplace research as both an expression of identity (eg, through personal items) and a tangible practice of desk ownership (eg, having the same workspace across days) (29, 30); (v) *teamwork*, reflecting the role of the physical environment in enabling collaboration and knowledge sharing (31, 32); (vi) *social interaction*, linked to both positive and negative consequences for workplace dynamics, but consistently highlighted as a driver of workplace appeal (10); and (vii) *number of days expected at work*, reflecting the growing importance of hybrid and flexible working, where office attendance depends on perceived affordances of both home and office environments (16, 33). Together, these attributes capture both spatial and psychosocial dimensions of the office environment and allow for analysis of employee office design preferences in hybrid work contexts.

**Table 1:** Workspace attributes and levels selected for the DCE.

Attribute	Description	Level
Office type/ design	The physical work environment regarding the office plan layout and functional features. 1. Cell-office. Own office room, with own printer or shared printer with colleagues 2. Shared office space (2–9 persons/open office space)* Personal workstation in shared workspace, often no printer within the space. Instead, resources such as printers and meeting rooms are shared with other colleagues in the office. Most of the work is performed at the personal workstation. 3. Medium-large office landscape (˃9 persons/open office space)* Personal workstation in shared workspace, often no printer within the space. Instead, resources such as printers and meeting rooms are shared with other colleagues in the office. Most of the work is performed at the personal workstation. 4. Activity-based office. An umbrella term that includes different activity-based office types. The office environment supports different office activities, and the workplace is chosen based on this (for example, for concentrated work individually and in groups, meetings, telephone calls, etc.). You can have both a flex space (not a personal space) or a personal space in this office type. One chooses a workplace according to the activity within the office. *= Combination of “shared room” 2–3 persons/room & “small office landscape” 4–9 persons/room	1. Cell-office 2. Shared office space 3. Medium-sized to large office landscape 4. Activity-based office
Desk sharing	Number of employees sharing a workstation (desks equipped with screen(s), keyboard, and mouse).	1. 0 (share with none) 2. 1–2 colleagues 3. More colleagues (no dedicated desk)
Privacy & quiet workspace	Separate space where employees can retreat for, for example, highly concentrated work, telephone conversations, digital meetings, etc.	1. Not at all possible 2. Limited possibility 3. Somewhat possible 4. A great deal possible
Personalization & territoriality	A sense of identity and desk ownership (i.e., the same workspace on consecutive days with the option to leave personal items regardless of office type).	1. Not at all possible 2. Limited possibility 3. Somewhat possible 4. A great deal possible
Teamwork	Workspaces that support teamwork - work in groups, meetings, and discussions	1. Not at all possible 2. Limited possibility 3. Somewhat possible 4. A great deal possible
Social interaction	Opportunity for social contacts and a network of interaction between employees in the organization.	1. Limited possibility 2. Somewhat possible 3. A great deal possible
Number of days expected at work (office)	The number of days the employee is expected to work in the ordinary office	1. 1 day 2. 2 days 3. 3 days 4. 4–5 days

### Study population

Participants were drawn from the Future Work project, which followed the reorganization of the working arrangement at this higher education institution. All employees in operational support roles (gross sample: N=1072) were invited to participate in the August 2021 baseline survey, and 862 responded (response rate: 81%), forming the panel that received the follow-up questionnaires. Of these, 3 dropped out, resulting in 859 respondents who received the DCE survey, and 485 employees completed it, with 433 providing full responses suitable for analysis. Participation was voluntary and uncompensated, with the option to withdraw at any time. Written informed consent was obtained at baseline for both the primary and DCE surveys. Data were pseudonymized and serially numbered to ensure anonymity throughout the study. A reminder was sent to non-respondents one week after the initial survey distribution.

### Survey instrument

A pretest of the survey instrument was conducted online with a convenience sample of 20 researchers and administrative staff from this higher education institution. This pretest assessed the comprehensibility of the DCE survey, the relevance and comprehensiveness of the attributes, the appropriateness of descriptive information, and the difficulty of the DCE questions for the target population. The pretest aimed to confirm participants' understanding of the attribute and level definitions. Open-ended responses indicated that participants clearly understood the attributes and levels.

The survey included seven office workspace attributes, each with three or four levels. The number of attributes and levels allows for many unique office workspace options. However, presenting every possible combination to respondents was impractical. For this reason, a random and statistically efficient non-orthogonal design, based on recommended design principles and practice in DCE (22), ensured that each level had the likelihood of appearing several times with minimal level overlap. The choice tasks were randomly created for main effects estimation from the attribute using the Ngene software for designing choice experiments (34). Participants were presented 12 questions on hypothetical office workspaces, each with two alternative answer options, and were asked to select their preference. No opt-out option was provided. A sample choice question is shown in table 2.

**Table 2:** An example of a choice question.

Attributes	Alternative 1	Alternative 2
Office type/ design	Activity-based office	Cell-office
Days at the office	2 days	3 days
Privacy & quiet space	Great deal possible	Somewhat possible
Personalization & territoriality	Somewhat possible	Great deal possible
Desk sharing	1–2 colleagues	0 (share with no one)
Teamwork	Somewhat possible	Limited possibility
Social interaction (relations)	Somewhat possible	Great deal possible
Which option would you choose?	☐	☐

### Statistical analysis

The data from the DCE survey were analyzed using a random parameters logit (RPL) model, following good research practice guidelines (21, 35–38). RPL models compared the office workspace preferences of each respondent to the different features of each workspace option in the choice questions, and thus, determined preference-weight estimates of each attribute and level included. Preference weights estimated from an RPL model indicated the relative strength of preference for each attribute level included in the survey; more-preferred outcomes had higher preference weights. These preference weights were used to calculate the importance of each attribute, conditional on the range of levels considered and relative to all other attributes included in the survey (21, 39). By estimating a distribution around each mean preference parameter, RPL models mitigated potential estimation bias in the mean preference-weight estimates that may have occurred because of unobserved preference heterogeneity among respondents (35, 36). The conditional relative importance (CRI) of each attribute was calculated as the difference in preference weights for the most- and least-preferred level of that attribute. The results were rescaled so that all CRI estimates summed to 100, and each CRI estimate was a proportion of 100.

Convergence was not achieved in the initial RPL model due to multicollinearity within attribute levels related to: (i) office type/design (including cell-office, shared office, medium-to-large office landscape, and activity-based office); (ii) desk sharing involving 0 (share with no one), 1–2, >2 colleagues (no dedicated desk); (iii) privacy and quiet workspace as well as personalization, territoriality and teamwork (ranging from "not at all" to "a great deal" possible); (iv) social interaction (limited possibility to a great deal possible); and (v) the number of days expected at work (ranging from 1, 2, 3, and 4–5 days). To mitigate multicollinearity, a composite level (variable) was created by aggregating the levels for these factors. While the RPL model effectively manages unobserved heterogeneity in preferences, it falls short of identifying observable characteristics linked to variations in work setting preferences (40). However, subgroup analysis allowed exploration of observed preference heterogeneity. This study conducted subgroup analysis for predefined, mutually exclusive subgroups, reporting only those with statistically significant preference differences based on distance, gender, age, household composition, tenure, office environment rating, home office rating, work arrangement (fixed or flexi), and home office disturbance. For each mutually exclusive set of subgroups in the sample, we created a dummy variable equal to 1 if the respondent belonged to the subgroup and interacted the dummy variable with each of the explanatory variables (attribute levels). The parameter on each of these interaction terms is interpreted as the difference between the subgroup and the corresponding attribute level. Differences in preferences between subgroups were tested through a log-likelihood test of joint statistical significance of all the interaction terms (P<0.05). A Wald test was used to determine the statistical significance of differences between adjacent attribute levels (P<0.05) for each attribute.

## Results

### Characteristics of respondents

The DCE survey elicited responses from 485 individuals out of the 859 invited, resulting in a response rate of 56%. Within this respondent cohort, 369 individuals identified as female, representing 76% of the total respondents. The average age of the respondents was 51 years (see table 3 for detailed information).

**Table 3:** Descriptive data on survey participants (N=485). [SD=standard deviation.]

		Mean (SD)	Frequency (%)
Age		51 (9.2)	
Weekly working hours		40 (5.2)	
Gender (female)			369 (76)
Managerial (No managerial position)			417 (86)
Household			
	Live alone or with an adult		202 (42)
	Live with another adult and/or child		279 (56)
Remote work current - 2023 (%)			
	No remote work (currently at 0)		19 (4)
	1–20		84 (17)
	21–49		268 (55)
	50–80		89 (18)
	>81		24 (5)
Remote work pre-COVID-19 (%) ^1^			
	No remote work (0 pre-COVID-19)		213 (44)
	20–100		252 (52)
Travel mode in the last week ^2^			
	Walked		40 (8)
	Biked		48 (10)
	Drove car		124 (26)
	Public transport		253 (52)
	Worked from home		17 (4)
	Absent last week		3 (0.6)
Travel distance (km) ^3^			
	<5–10		205 (43)
	>10		280 (57)
Employment type			
	Permanent		462 (96)
	Temporary		18 (4)
Work schedule ^4^			
	Fixed		204 (42)
	Flextime		263 (54)
Years of work at the organization			
	<1–5		207 (43)
	>5		273 (57)
Office type pre-COVID-19 (2020) ^5^			
	Cell		187 (39)
	Shared (2–3 people)		132 (27)
	Shared (4–9 people)		78 (16)
	Shared (10–24 people)		23 (5)
	Open plan (≤25 people)		29 (6)
	Hot-desking (use a free desk)		1 (0.2)
	Activity-based		3 (0.6)
	Remote work only		10 (2)
	Other		14 (3)
Office type (2023)			
	Cell		186 (38)
	Shared (2–3 people)		103 (21)
	Shared (4–9 people)		74 (15)
	Shared (10–24 people)		24 (5)
	Open plan (≤25 people)		22 (5)
	Hot-desking (use a free desk)		4 (0.8)
	Activity-based		4 (0.8)
	Remote work only		58 (12)
	Other		3 (0.6)
Office rating ^6^			
	Somewhat good		152 (31)
	Very good		318 (66)
Home office rating ^6^			
	Somewhat good		240 (50)
	Very good		104 (48)
Disturbed at home office ^7^			
	Strongly disagree		233 (48)
	Somewhat agree		171 (35)

^1^ Data used from baseline survey, asking participants to think back on how much they worked remotely before the COVID-19 pandemic. ^2^ If multiple modes, choose the mode used for the longest part of the travel. ^3^ Distance between home and office, one way. ^4^ Work schedule, data used from baseline survey. ^5^ Office types before the pandemic, data used from the baseline survey. ^6^ Regular office / home office physical work environment rating. ^7^ Often disturbed when working from home (baseline survey data).

### Preference weights and conditional relative importance of workspace attributes

**Figure 1:**
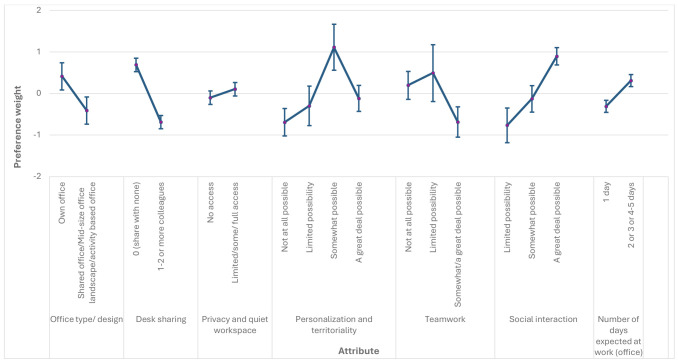
Attribute relative importance change (N=433). Note: The vertical bars surrounding each mean preference weight denote the 95% confidence interval of the point estimate (preference weights computed by the delta method).

Of the 485 DCE survey respondents, 433 fully completed the choice questions. Figure 1 shows the mean preference-weight estimates and 95% confidence intervals (CI) for each attribute level. These relative preference weights indicate the comparative desirability of attribute levels. As shown in the sequence presented in the DCE questions, moving from shared, mid-size, or activity-based offices to cell offices significantly increased preference strength, demonstrating that employees favored personal workspaces. Similarly, having one's own private desk was more strongly preferred than desk sharing. Privacy and quiet workspace availability did not significantly affect preferences. Personalization and territoriality were positively valued, with higher levels more strongly preferred. Enhanced opportunities for collaboration and social interaction were also preferred. Finally, the expected number of office workdays showed a negative association with preferences, suggesting employees were most favorable toward arrangements with ≥2 days in the office.

**Figure 2:**
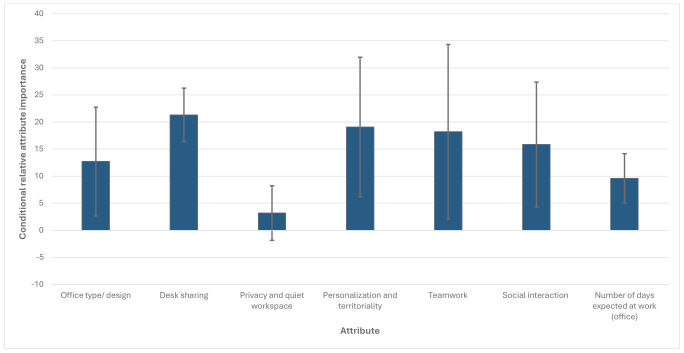
Conditional attribute relative importance (N=433). Note: The conditional relative importance is the difference between the preference weights on the most and least influential attribute levels. These differences are summed across attributes, and the sum is scaled to 100. The conditional importance of each attribute is a percentage of this total. The vertical bars surrounding each relative importance weight estimate denote the 95% confidence interval around the point estimate (computed by the delta method).

Figure 2 illustrates the CRI of each attribute. Desk sharing had the highest CRI, indicating it was the most important attribute, followed by personalization and territoriality, teamwork, social interaction, office type/design, number of days expected at work/office, and privacy and quiet workspace. Privacy and quiet space were not significant environmental factors (P=0.213). See also supplementary material, www.sjweh.fi/article/4264, tables S1 and S2, for detailed results.

### Subgroup analysis

Table 4 presents subgroup analyses of office workspace preferences. Commuting distance, gender, age, household composition, tenure, office and home environment ratings, working arrangement, and home office disturbances significantly affected these preferences. Longer commutes correlated with prioritization of personalization, social interaction, and desk sharing, while shorter commutes emphasized desk sharing. Personalization was important for both genders, but teamwork was less so for men. Younger respondents (≤50 years) valued personalization and low levels of desk sharing, while older respondents prioritized social interaction. Individuals living alone or with only adults valued social interaction more than those living with children and/or other adults. Newer employees prioritized personalization, while longer-tenured employees focused on teamwork. A 'somewhat good' home office environment shifted the focus to social interaction, while a 'very good' home environment emphasized personalization. Flexible schedules correlated with personalization, and fixed schedules with social interaction. Finally, home office disruptions led to a higher valuation of personalization, while no disruptions correlated with teamwork. Supplementary figures S1–9 (subgroup analysis of office workspace preferences) visually represent the results in table 4.

**Table 4:** Subgroup analysis of office workspace preferences.

Subgroup set & sample size	Summary of results
**Distance (km)** <5–10 (N=205) >10 (N=280) P-value=0.009	Distance was a key factor influencing preferences for office workspace. For those commuting >10 km, personalization and territoriality were top priorities, followed by desk sharing and social interaction, teamwork, and the number of workdays in the office. Office type and availability of privacy and quiet workspace were not significant in their decision-making. In contrast, respondents with commutes of 5–10 km valued desk sharing the most, followed by social interaction, personalization, and the number of workdays in the office. Teamwork, office type, and private and quiet workspace did not influence their choice of office space, as depicted in supplementary figure S1.
**Gender** Female (N=369 Male (N=110) P-value=0.007	Gender influenced office workspace preferences among respondents. For women, personalization and territoriality were top priorities, followed by social interaction, desk sharing, office type, teamwork, workdays in the office, and privacy. Teamwork and having a private, quiet workspace did not impact their preferences. Men showed a broadly similar ranking of attributes, also prioritizing personalization and territoriality. However, while the conditional importance of teamwork appears relatively pronounced in supplementary figure S2, the underlying preference weights did not reach statistical significance. This suggests that teamwork was less consistently influential for men compared to women. Similarly, workdays in the office, office type/design, and privacy/quiet workspace availability did not significantly shape men’s choices when confidence intervals were considered.
**Age (years)** ≤50 (N=214) ≥51 (N=266) P-value=0.001	The respondents’ age was a significant factor in determining office workspace preferences. Those aged ≤50 and ≥51 valued personalization and territoriality the most. Those aged ≤50 prioritized the attributes of personalization, desk sharing, social interaction, teamwork, and the number of workdays in the office, in that order. However, respondents ≥51 prioritized personalization, desk sharing, social interaction, followed by teamwork, the number of workdays in the office, and privacy and quiet workspace. Teamwork was not a significant factor for those ≥51 in their office workspace choices. Both age group respondents showed little concern for office type and the availability of privacy and quiet workspace in their preferences, as shown in supplementary figure S3.
**Household composition** Live alone or with an adult (N=202) Live together with kids and/or an adult (N=279) P-value=0.019	Household composition significantly influenced office workspace choices. Respondents living alone consider social interaction as the most important attribute. This was followed by personalization, desk sharing, and the number of workdays in the office. Those living with kids and/or an adult valued personalization and territoriality most, followed by social interaction, desk sharing, and the number of workdays in the office. Office type did not impact office workspace choice for those living with kids and/or an adult, while teamwork and privacy did not affect preferences for both groups (see supplementary figure S4).
**Years of work (tenure)** <1–5 (N=207) >5 years (N=273) P-value=0.003	The length of employment had a significant impact on office workspace preferences. Individuals with <5 years of tenure prioritized personalization and territoriality, then social interaction, office type, desk sharing, and the number of workdays in the office. On the other hand, those with over 5 years valued teamwork the most, followed by social interaction, desk sharing, personalization, and the number of workdays in the office. However, office type did not affect the choice of work setting for respondents with >5 years of tenure, and privacy did not impact preferences for either group, as shown in supplementary figure S5.
**Rating of the regular office environment** Somewhat good (N=152) Very good (N=318) P-value=0.001	The rating of the regular office environment significantly influenced respondents’ office workspace preferences. Those who found the office environment ‘somewhat good’ prioritized personalization and territoriality, then social interaction, desk sharing, office type, workdays in the office, and teamwork. However, privacy and a quiet workspace did not impact their choices in work settings. On the other hand, respondents who rated the office environment as very good saw social interaction as the most important factor. They then considered personalization and territoriality, desk sharing, and workdays in the office. However, teamwork, office type, and privacy and quiet workspace did not influence their preferences for office workspace, as shown in supplementary figure S6.
**Rating of home office environment** Somewhat good (N=240) Very good (N=233) P-value <0.001	Those who rated their home office as somewhat good valued social interaction the most, desk sharing, and a private and quiet workspace. However, teamwork, office type, personalization and territoriality, and the anticipated workdays did not affect their choices. On the other hand, respondents who rated their home office as very good considered personalization and territoriality as the most important features. Social interaction, desk sharing, teamwork, office type, and workdays in the office were ranked in descending order of importance. However, the presence of privacy and a quiet workspace did not impact their office workspace preferences, as shown in supplementary figure S7.
**Working arrangements** Fixed schedules (N=204) Flextime (N=263) P-value=0.016	The different working arrangements were found to be key factors influencing respondents’ choices for office workspace. For those on fixed schedules, social interaction was paramount, followed by personalization and territoriality, desk sharing, and workdays in the office. However, teamwork, office type, and privacy and quiet workspace did not sway their preferences. Conversely, individuals with flexible hours valued personalization and territoriality the most. Subsequently, social interaction, desk sharing, teamwork, office type, and workdays in the office were considered in decreasing order of importance. However, teamwork and the presence of privacy and quiet workspace did not affect their choices, as shown in supplementary figure S8.
**Disturbances in the home office** Somewhat agree (N=171) Strongly disagree (N=233) P-value <0.001	The impact of disruptions in the home office has been identified as a key factor affecting office workspace preferences. Those who ‘somewhat agreed’ that they experience disruptions at home valued personalization and territoriality the most, followed by social interaction, desk sharing, office type, and workdays in the office. Teamwork and having privacy and a quiet workspace did not impact their choices of office workspace. On the other hand, individuals who ‘strongly disagreed’ that they experience disruptions at home considered social interaction as the most crucial aspect. Subsequently, personalization and territoriality, and desk sharing were ranked in decreasing order of importance. However, this group showed indifference towards the impact of teamwork and privacy and quiet on their work environment preferences, as shown in supplementary figure S9.

## Discussion

This DCE survey investigated how specific office components impact workspace preferences of operational support employees at a higher education institution, focusing on office design, desk sharing, privacy, personalization, teamwork, social interaction, and required on-site days.

The results indicated a preference for desk ownership and personalized workspaces, with opportunities to have a sense of identity and the same workspace on consecutive days, with the option to leave personal items and equipment, teamwork, and social interaction. The social spaces were preferred because they provide relaxation and space for informal meetings, while teamwork spaces, both physical and digital, facilitate collaboration through group work, meetings, and discussions.

The findings highlight some salient workspace attributes. The study emphasizes that employees prefer to have little or no desk sharing, personalization, and opportunities for social interaction within the workspace. Personalization satisfies the need for personal space and self-identity, for instance, by enabling consistent seating or the use of personal markers such as family photos (30). Strong social relationships at work, in turn, contribute to a positive psychosocial environment (31). Our findings suggest that employees perceive the physical workspace as an important facilitator of such relationships. We acknowledge that prior research has documented the complexity of these associations, showing that environmental features can have both positive and negative impacts on social interaction depending on their configuration and use [eg (24, 27),]. For instance, employees reported lower productivity when their office design failed to deliver on interaction and personalization, with the effect being more pronounced in non-territorial offices than territorial ones (24). By focusing on employee preferences, our study adds to this body of work by highlighting the perceived importance of spaces that allow for social connection, even though the actual behavioral outcomes of such designs were not directly assessed here.

The strong preference for territoriality and personal desks observed in this study aligns with previous findings that emphasize the importance of identity and stability in shared office settings. At the same time, research suggests that non-territorial offices can bring advantages when multiple employees share workspaces, including improved space use and organizational economic benefits (24, 27). As Kim et al (24) found, it is not desk ownership per se that determines satisfaction and productivity, but rather whether the spatial environment meets core employee needs such as opportunities for interaction, personalization, and adequate storage. This highlights the need for caution when translating user preferences directly into design practice. While preference studies highlight employees' perceived needs, preferred features may also carry unintended trade-offs. For example, territorial designs may strengthen identity and comfort but could reduce space efficiency, hinder flexibility, or exacerbate vacancy rates in a hybrid work context. Non-territorial designs can amplify the importance of design quality such that if interaction or personalization falls short, employees in desk-sharing contexts feel it more strongly than those with allocated desks. Thus, workplace design decisions should balance employees' preferences with broader organizational, spatial, and sustainability considerations to ensure that short-term satisfaction does not come at the expense of long-term functionality.

While some opportunity for teamwork was valued (positive coefficient for “limited possibility”), very high levels of teamwork were not associated with higher preferences and, in fact, reduced preference relative to the reference category. This pattern is consistent with earlier findings suggesting that employees value a balance between collaboration and opportunities for focus, rather than maximizing teamwork alone [eg (10, 41),].

The findings indicate that additional quiet spaces were not the preferred strategy for achieving privacy, which should not be interpreted as undermining the value of privacy overall, especially given that private offices remained a preferred option. While privacy is typically recognized as a central aspect of office design, encompassing acoustic, speech, and visual dimensions (42), our study captured privacy specifically in terms of access to additional quiet rooms. This narrower framing could partly explain why quiet areas were not prioritized, as prior research shows that employees are often reluctant to switch workspaces [eg (12, 13),]. Moreover, many respondents in this sample already had access to shared or private rooms, which could have reduced the perceived need for additional quiet spaces. Thus, rather than contradicting the general importance of privacy in the workplace (43, 44), our findings suggest that, in this context, employees may prefer to achieve privacy through existing spatial provisions or within their own personal workstations, rather than through separate designated quiet areas.

Employee preferences for work settings vary significantly based on factors such as commute distance, gender, age, household composition, tenure, office environment ratings, home office ratings, working arrangement, and home office distractions. This aligns with prior research showing gender differences in office feature preferences; for instance, men in activity-based offices reported greater dissatisfaction with desk sharing and personalization limitations (7) and were more prone to sick leave (45). Employees with longer commutes (≥10 km) prioritized personalized workspace, social interaction, and teamwork, while those with shorter commutes preferred having their own desk first (not sharing), followed by teamwork and social interaction. Long-tenured employees (≥5 years) valued social and networking opportunities, whereas newer employees preferred a separate, personal office. This is particularly relevant for organizations with new recruits or high turnover. These findings underscore the importance of considering employee characteristics and experiences when implementing changes to future office spaces.

While an increased inclination to remote work was anticipated post-COVID-19, understanding individual preferences for returning to the office remains unclear. Our analysis of the desired in-office days shows a preference for ≥2 days in the office, identifying employee groups more inclined to return than to work remotely (17). The findings from this study suggest that the number of desired in-office days can segment employees based on workspace preferences, indicating a need for tailored solutions rather than a universal approach to workplace flexibility. This segmentation raises complexities beyond current remote work trends.

### Strengths and limitations

The DCE is a rigorous methodology using surveys, piloting, and an experimental design developed using established research practices. The study used advanced RPL methods to analyze office workspace-choice data, mitigating estimation bias from unobserved preference variations and within-sample correlation. This DCE study uniquely analyzes environmental factors influencing office work environment preferences. The relative preference information from the DCE is readily applicable to design practice and policy implementation. Given the influence of office design and expected days in the office on workspace choices, and subsequently on worker productivity and health, organizations should consider specific workspace design components that influence preferences when recommending changes to the physical work environment.

This study has several limitations. Firstly, the sampling procedure was based on a full census of the target employee group within the institution. However, self-selection bias remains a possible limitation, as participation was voluntary, and individuals with stronger opinions or interest in workplace issues may have been more likely to respond. Secondly, the predominantly female sample (76%) may bias results as gender can influence office environment preferences. Thirdly, the hypothetical nature of the scenarios may not accurately reflect real-world workspace decisions, potentially oversimplifying complex decision-making processes when employees are presented with actual office workspaces. A potential limitation is also the framing of attributes, particularly the inclusion of both symbolic and concrete elements in the definition of personalization and territoriality, which may have influenced the strength of stated preferences. Future research could explore how the alternative framing of attribute descriptions affects preference outcomes. Furthermore, the data were collected when COVID-19-related policies and concerns were still prevalent, which may have influenced preferences for office attendance and social interaction.

In addition to these limitations, the broader work environment context must be considered. Most respondents worked in traditional, territorial offices, which may have made it easier and more reliable for them to evaluate familiar features compared to hypothetical alternatives. This likely enhanced the validity of their stated preferences as these were based on everyday experience rather than speculation. At the same time, the ongoing debate about high office absenteeism after the pandemic and the potential introduction of more flexible office designs may have influenced respondents' views. At the time of the survey, the organizations were in the process of implementing hybrid work arrangements, but no concrete plans for physical office redesign had been communicated. It is therefore possible that respondents' preferences were partly shaped by concerns related to hybrid work policies (eg, increased remote work or changes in attendance patterns) or expectations of physical workspace redesign. However, the lack of concrete redesign plans suggests that the reported preferences largely reflect their evaluations of existing territorial offices. These limitations warrant cautious interpretation and consideration of the broader applicability of the findings. Future research should explore evolving workspace preferences across contexts and conditions in the post-pandemic era.

Notwithstanding this, the study highlights relevant findings for organizations adapting their offices for flexible work, as well as property owners and professionals involved in creating office environments (eg, architects/designers, facility managers). It can be challenging to prioritize certain environmental factors in hybrid workplaces where the choice must be made between different office characteristics, as not all employees' wishes can be satisfied. Thus, empirical knowledge is crucial for informed decision-making, especially given resource constraints and the need for efficiency. Further research is needed to optimize hybrid office environments as this issue concerns not only supporting employee needs but also aiding recruiting and retaining skilled workers in a competitive labor market.

The sample in this study is drawn from administrative personnel within a higher education institution, possessing specific organizational characteristics. Employment within the public sector differs significantly from that in the private sector, and working in a large organization, such as the one in this study, is distinct from working in a smaller one. Moreover, work environments and employee situations vary considerably depending on the line of business. Administrative work in an educational organization differs from administrative work in organizations within innovative or high-tech sectors. Therefore, while our findings are directly generalizable to operational support staff in this institution, caution is warranted when extrapolating to other occupational groups or organizational contexts.

### Study implications

This study advances understanding of post-pandemic office design by demonstrating how physical workspace features are closely linked to preferences for workspaces, especially spaces for social interaction, and features that encourage presence in the office in a FWA context, eg, preference for desk sharing arrangements. For instance, our finding that employees strongly value opportunities for social interaction and teamwork in the office highlights a central challenge for post-pandemic workspace design. This can be critical for how to effectively foster these valued social connections within FWA. While pre-pandemic research established that spatial design, visibility, and proximity can facilitate communication and team identity (46, 47), our study underscores their perceived importance to employees in a context where everyday office attendance is no longer mandatory. This suggests that the office's new role may be primarily social and collaborative. However, this design goal creates a critical tension. As our results also show, employees simultaneously value control over their personal workspace (eg, dedicated desks, personalization) to mitigate distractions. Therefore, the implication for practice is not simply to create more open, interactive spaces but to strategically balance these with provisions for psychological comfort and concentration (43, 48). A key question for future research in the hybrid context is how to design environments that successfully integrate spaces for spontaneous interaction without exacerbating the environmental stressors that can make the home office seem more attractive for focused work. In line with this, some research has found that large, shared workspaces can lead to less face-to-face communication and an increase in electronic interaction between colleagues (49). Given the central role of communication and social relationships for both employee and organizational well-being and performance, further research is needed in a hybrid work context to explore the dynamics of closely collocated teams versus those that are physically separated.

The findings also highlight the importance of considering workforce composition when implementing workplace design changes, as preferences vary based on demographics and location. While remote work has been studied, the post-pandemic shift requires further investigation to optimize the setup of new office landscapes. Future studies should investigate the impact of various office designs on flexible work practices, the significance of employee involvement, and cost-effectiveness for employers, considering work performance, productivity, and operational efficiency.

### Concluding remarks

This study shows that operational support staff in higher education favor office designs in hybrid work contexts that provide a dedicated desk, allow for personalization, and facilitate teamwork and social interaction, with a preference for being on-site ≥2 days per week. The findings provide actionable knowledge for organizational decision-makers, facility managers, and occupational health and safety (OHS) professionals. For OHS practice, this means advocating for workspace designs that directly support employee well-being by mitigating environmental stressors and fostering positive psychosocial conditions. These findings can inform the design of flexible offices in higher education that are not only functional but also promote health, satisfaction, and sustainable work.

## Supplementary material

Supplementary material

## Data Availability

Subject to appropriate ethical and legal considerations, data can be shared for research purposes. Data requests can be sent to irene.jensen@ki.se.
