# Supplementary material for: Employee workspace preferences in a mandated hybrid work policy: A discrete choice experiment
Source: Scand J Work Environ Health. 2026 Feb 28;52(2):147–59. doi: 10.5271/sjweh.4264 (PMC12957959; doi:10.5271/sjweh.4264)
Supplement: Supplementary material [file SJWEH-52-147-S001.pdf]

Employee workspace preferences in a mandated hybrid work policy: A discrete choice experiment[1]

by Emmanuel Aboagye, PhD,[2] Willings Botha, PhD, Helena Tinnerholm Ljungberg, PhD, Christina Bodin Danielsson, PhD, Irene Jensen, PhD

1. Supplementary material
2. Correspondence to: Emmanuel Aboagye, Unit of Intervention and Implementation Research for Worker Health, Institute of Environmental Medicine, Karolinska Institutet, 171 77, Stockholm, Sweden. [E-mail: emmanuel.aboagye@ki.se]

S1 Table. Preference Weights (N=433)

**Attributes**	**Levels**	**Utility**	***P-Value***
Office type/ design	Own office	0.411	0.013
	Shared office/Mid-size office landscape/activity-based office	-0.411	0.013
Desk sharing	0 (share with none)	0.688	<0.001
	1-2 or more colleagues	-0.688	<0.001
Privacy and quiet workspace	No access	-0.103	0.213
	Limited or some access or full access	0.103	0.213
Personalization and territoriality	Not at all possible	-0.693	<0.001
	Limited possibility	-0.298	0.218
	Somewhat possible	1.112	<0.001
	A great deal possible	-0.12	0.452
Teamwork	Not at all possible	0.196	0.255
	Limited possibility	0.491	0.158
	Somewhat/a great deal possible	-0.686	<0.001
Social interaction	Limited possibility	-0.766	<0.001
	Somewhat possible	-0.129	0.43
	A great deal possible	0.895	<0.001
Number of days expected at work (office)	1 day	-0.31	<0.001
	2, 3 or 4-5 days	0.31	<0.001

S2 Table. Attribute Relative Importance Changes (N = 433)

**Attributes**	**From Level**	**To Level**	**Change in Utility (Difference Calculation)**	***p Value***
Office type/ design	Shared office/Mid-size office landscape/activity-based office	Own office	0.822	0.013
Desk sharing	1-2 or more colleagues	0 (share with none)	1.377	<0.001
Privacy and quiet workspace	Limited or some access or full access	No access	-0.207	0.213
Personalization and territoriality	Limited possibility	Not at all possible	-0.395	0.299
	Somewhat possible	Not at all possible	-1.805	<0.001
	A great deal possible	Not at all possible	-0.573	0.058
	Somewhat possible	Limited possibility	-1.41	0.006
	A great deal possible	Limited possibility	-0.178	0.233
	A great deal possible	Somewhat possible	1.232	0.004
Teamwork	Not at all possible	Limited possibility	-0.295	0.567
	Not at all possible	Somewhat/a great deal possible	0.882	<0.001
	Limited possibility	Somewhat/a great deal possible	1.177	0.027
Social interaction	Somewhat possible	Limited possibility	-0.638	0.078
	A great deal possible	Limited possibility	-1.662	<0.001
	A great deal possible	Somewhat possible	-1.024	<0.001
Number of days expected at work (office)	2, 3 or 4-5 days	1 day	-0.621	<0.001

Figure S1: Conditional Attribute Relative Importance – Distance

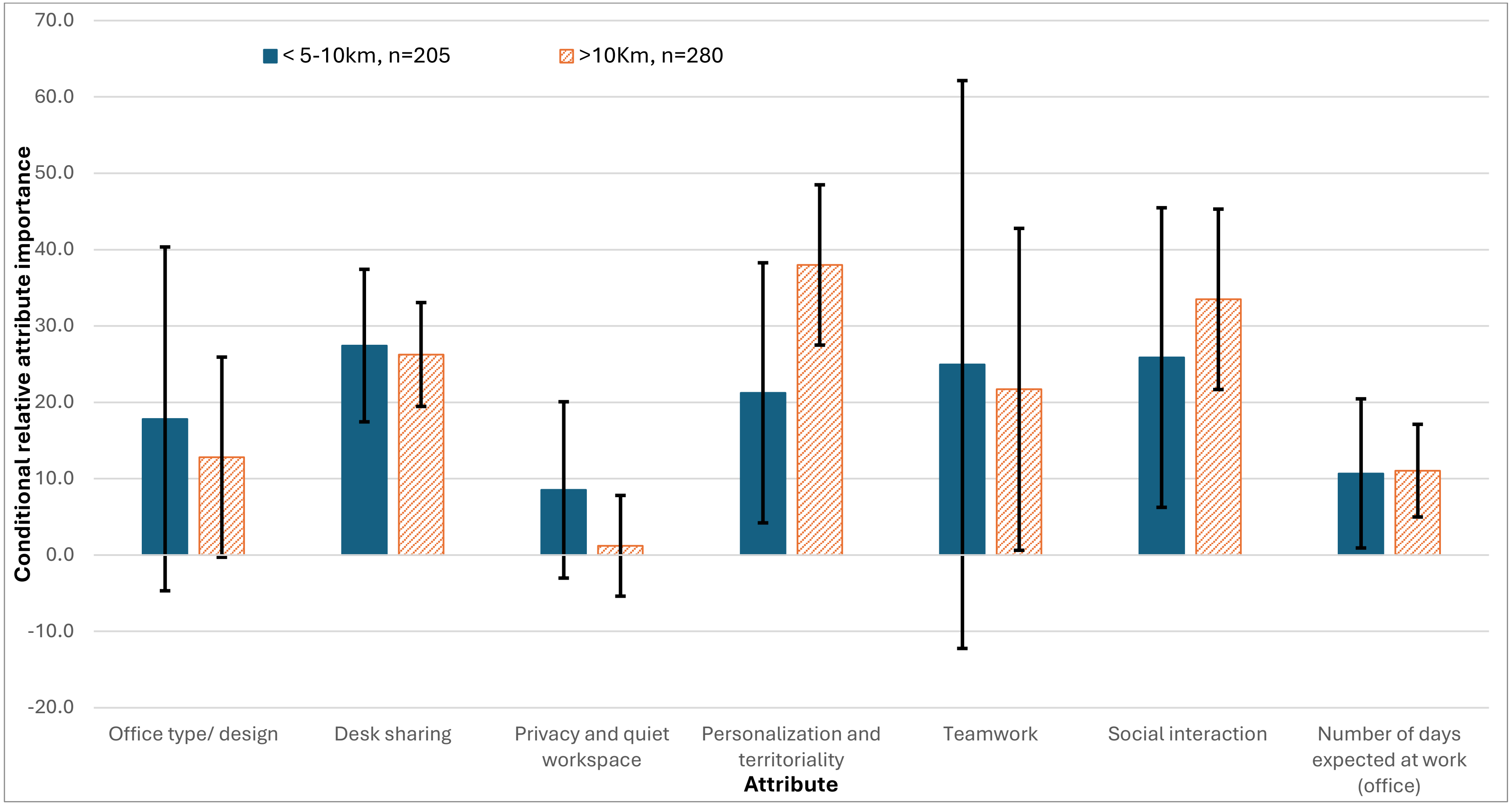

Note: The conditional relative importance is the difference between the preference weights on the most influential attribute level and the least influential attribute level. These differences are summed across attributes and the sum is scaled to 100. The conditional importance of each attribute is a percentage of this total. The vertical bars surrounding each relative importance weight estimate denote the 95% CI around the point estimate (computed by the delta method).

Figure S2: Conditional Attribute Relative Importance – Gender

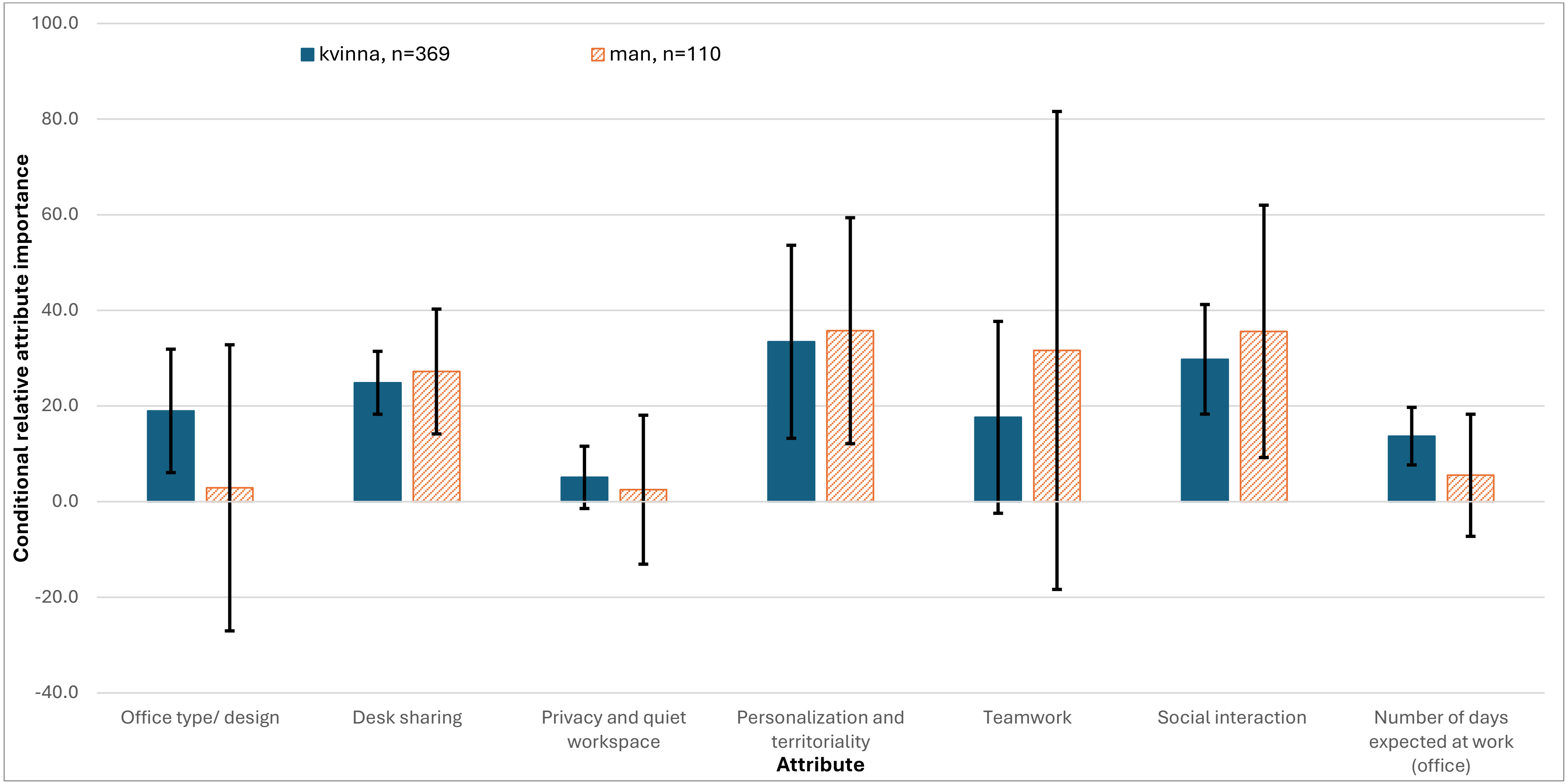

Note: The conditional relative importance is the difference between the preference weights on the most influential attribute level and the least influential attribute level. These differences are summed across attributes and the sum is scaled to 100. The conditional importance of each attribute is a percentage of this total. The vertical bars surrounding each relative importance weight estimate denote the 95% CI around the point estimate (computed by the delta method).

Figure S3: Conditional Attribute Relative Importance – Age

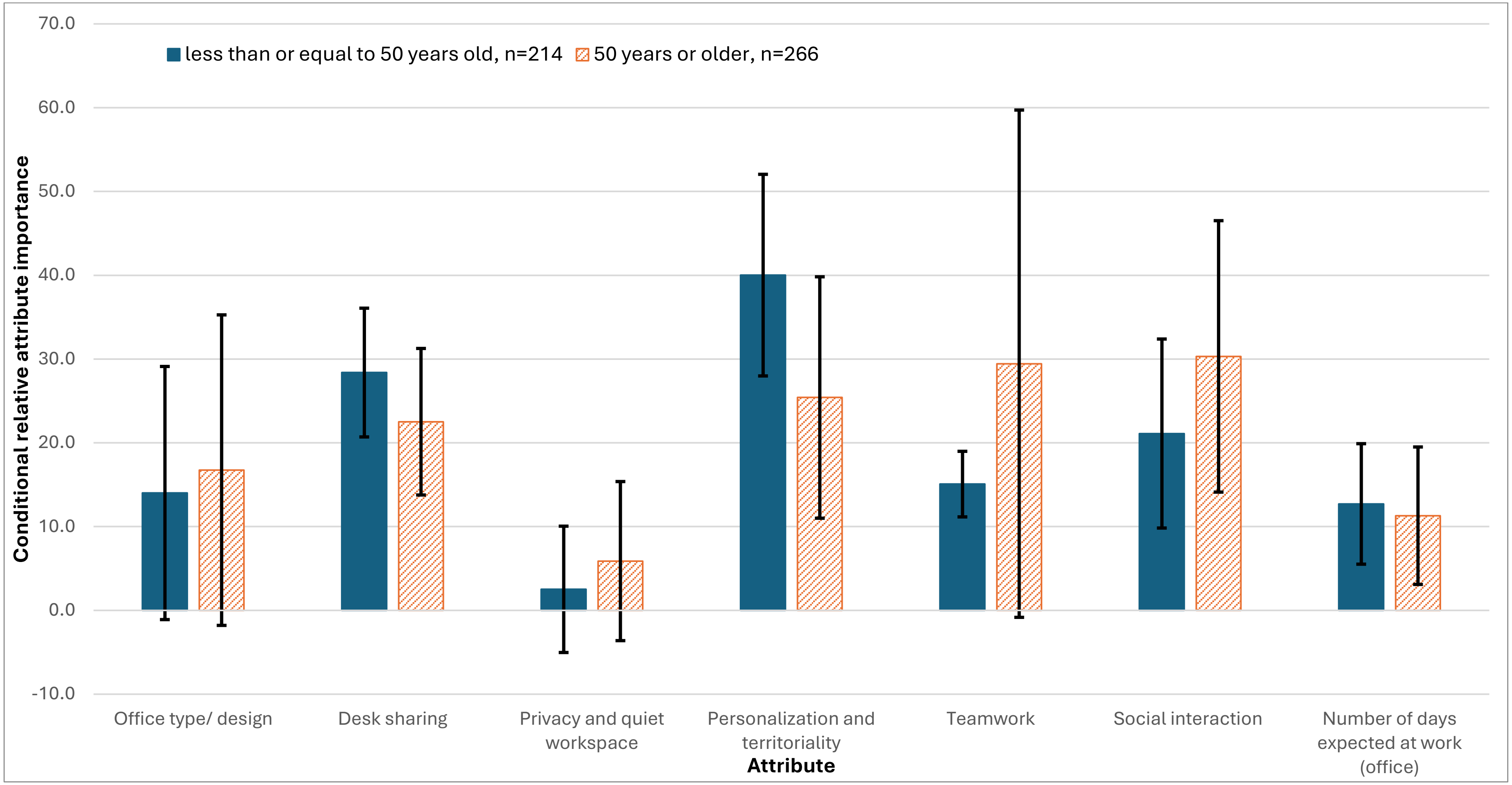

Note: The conditional relative importance is the difference between the preference weights on the most influential attribute level and the least influential attribute level. These differences are summed across attributes and the sum is scaled to 100. The conditional importance of each attribute is a percentage of this total. The vertical bars surrounding each relative importance weight estimate denote the 95% CI around the point estimate (computed by the delta method).

Figure S4: Conditional Attribute Relative Importance – Household composition

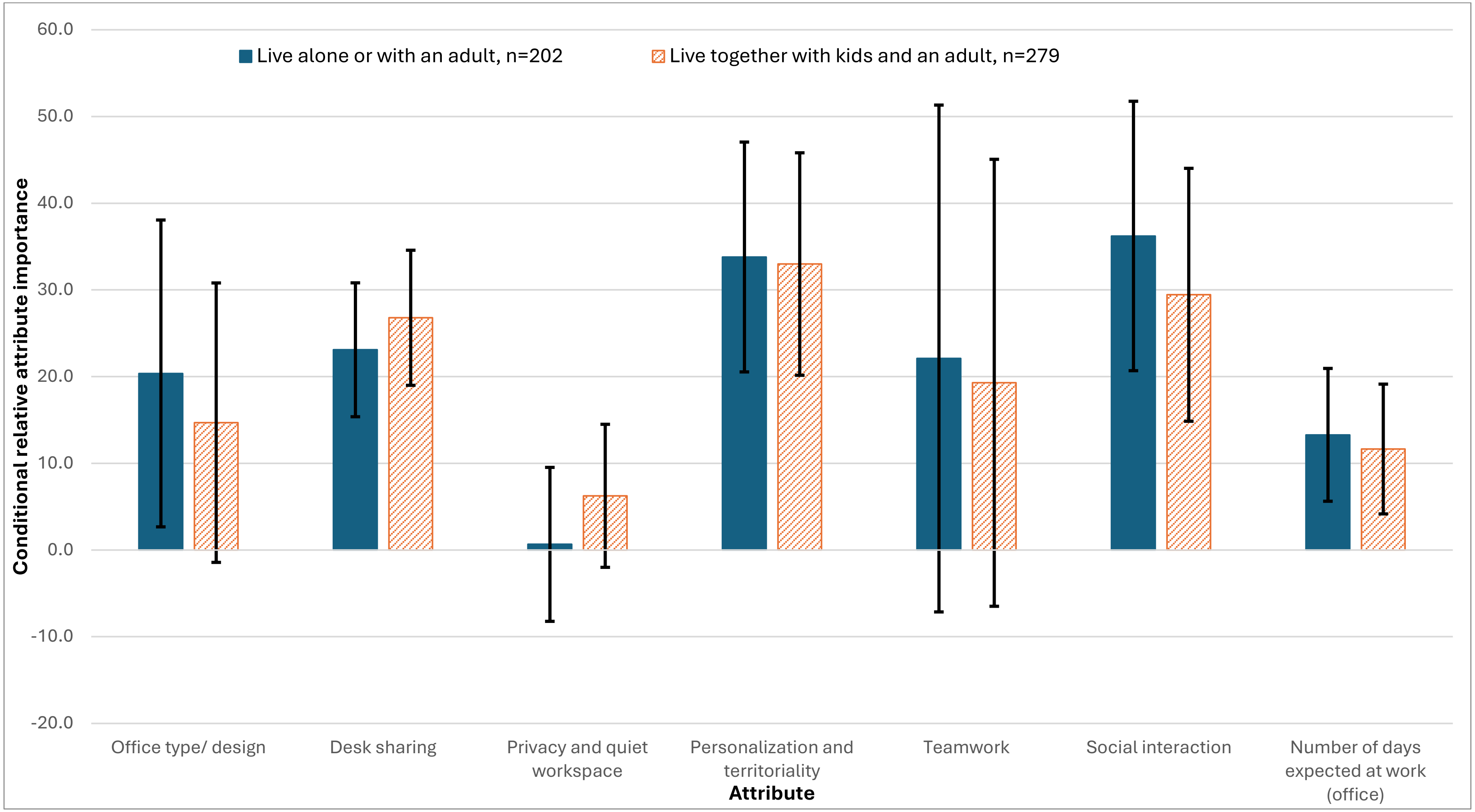

Note: The conditional relative importance is the difference between the preference weights on the most influential attribute level and the least influential attribute level. These differences are summed across attributes and the sum is scaled to 100. The conditional importance of each attribute is a percentage of this total. The vertical bars surrounding each relative importance weight estimate denote the 95% CI around the point estimate (computed by the delta method).

Figure S5: Conditional Attribute Relative Importance – Tenure

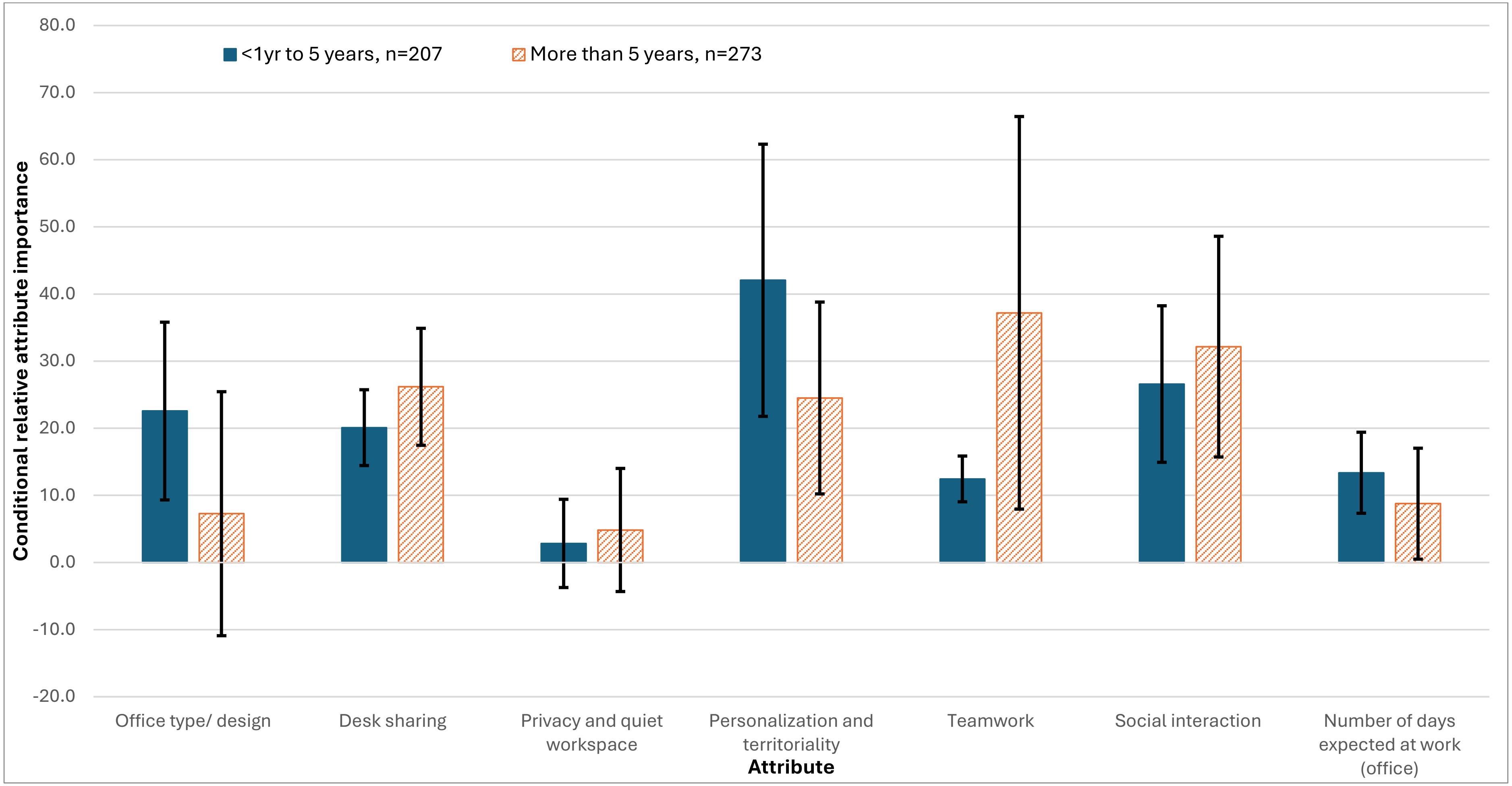

Note: The conditional relative importance is the difference between the preference weights on the most influential attribute level and the least influential attribute level. These differences are summed across attributes and the sum is scaled to 100. The conditional importance of each attribute is a percentage of this total. The vertical bars surrounding each relative importance weight estimate denote the 95% CI around the point estimate (computed by the delta method).

Figure S6: Conditional Attribute Relative Importance – Rating of employer's office environment

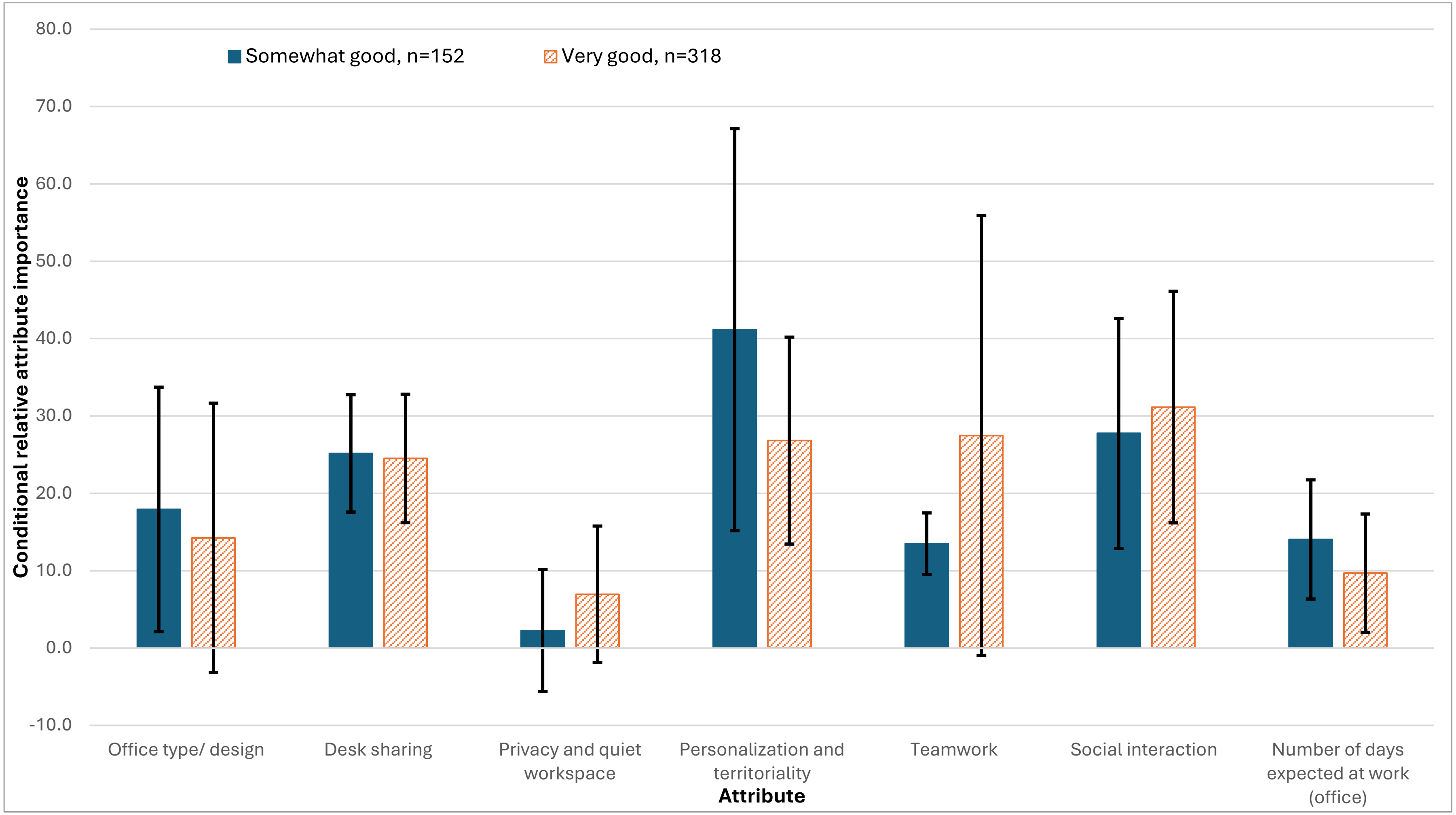

Note: The conditional relative importance is the difference between the preference weights on the most influential attribute level and the least influential attribute level. These differences are summed across attributes and the sum is scaled to 100. The conditional importance of each attribute is a percentage of this total. The vertical bars surrounding each relative importance weight estimate denote the 95% CI around the point estimate (computed by the delta method).

Figure S7: Conditional Attribute Relative Importance – Rating of home office

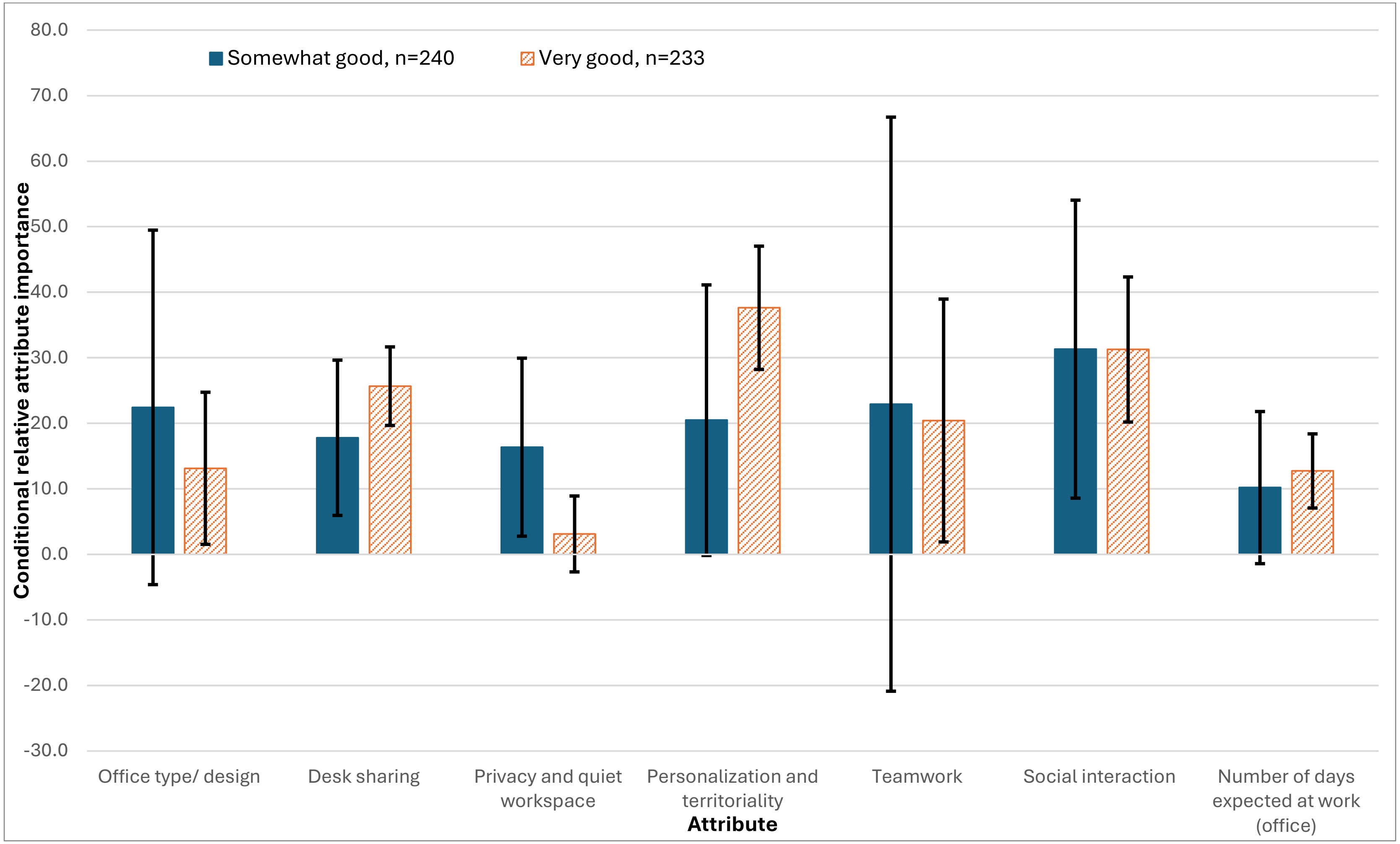

Note: The conditional relative importance is the difference between the preference weights on the most influential attribute level and the least influential attribute level. These differences are summed across attributes and the sum is scaled to 100. The conditional importance of each attribute is a percentage of this total. The vertical bars surrounding each relative importance weight estimate denote the 95% CI around the point estimate (computed by the delta method).

Figure S8: Conditional Attribute Relative Importance – Work form option

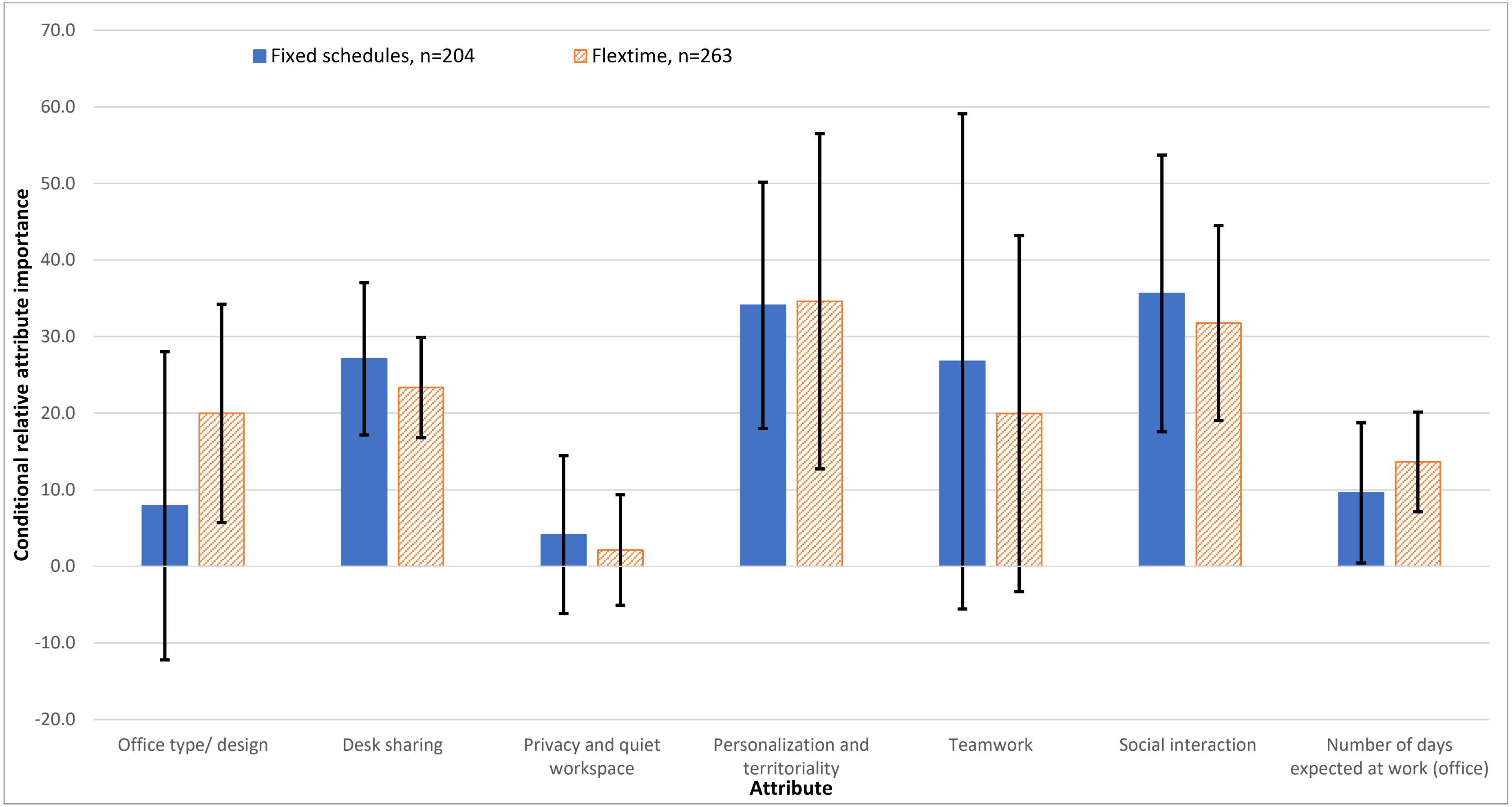

Note: The conditional relative importance is the difference between the preference weights on the most influential attribute level and the least influential attribute level. These differences are summed across attributes and the sum is scaled to 100. The conditional importance of each attribute is a percentage of this total. The vertical bars surrounding each relative importance weight estimate denote the 95% CI around the point estimate (computed by the delta method).

Figure S9: Conditional Attribute Relative Importance – Disturbance

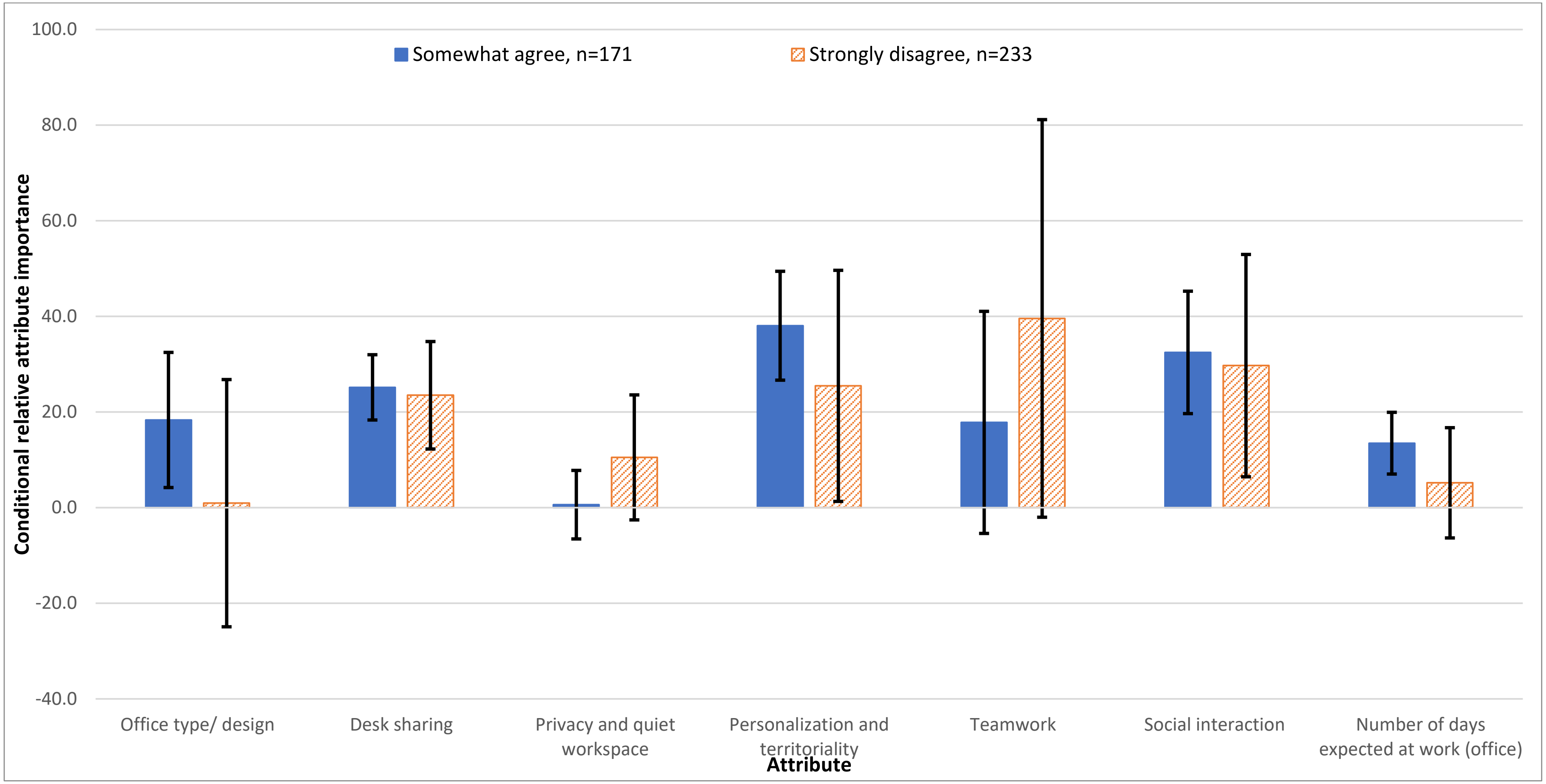

Note: The conditional relative importance is the difference between the preference weights on the most influential attribute level and the least influential attribute level. These differences are summed across attributes and the sum is scaled to 100. The conditional importance of each attribute is a percentage of this total. The vertical bars surrounding each relative importance weight estimate denote the 95% CI around the point estimate (computed by the delta method).
